# Structural Studies of the 3′,3′-cGAMP Riboswitch Induced by Cognate and Noncognate Ligands Using Molecular Dynamics Simulation

**DOI:** 10.3390/ijms19113527

**Published:** 2018-11-09

**Authors:** Chaoqun Li, Xiaojia Zhao, Xiaomin Zhu, Pengtao Xie, Guangju Chen

**Affiliations:** 1 College of Chemistry, Chemical Engineering and Materials, Handan University, No. 530 North Xueyuan Road, Hanshan District, Han Dan 056005, Hebei, China; zhaoxiaoya@hdc.edu.cn (X.Z.); libin@hdc.edu.cn (X.Z.); hdxyxpt@gmail.com (P.X.); 2 College of Chemistry, Beijing Normal University, 19# Xinjiekouwai Street, Beijing 100875, China; gjchen@bnu.edu.cn

**Keywords:** molecular dynamics simulation, 3′,3′-cGAMP riboswitch, allosteric communication, ligand recognition

## Abstract

Riboswtich RNAs can control gene expression through the structural change induced by the corresponding small-molecule ligands. Molecular dynamics simulations and free energy calculations on the aptamer domain of the 3′,3′-cGAMP riboswitch in the ligand-free, cognate-bound and noncognate-bound states were performed to investigate the structural features of the 3′,3′-cGAMP riboswitch induced by the 3′,3′-cGAMP ligand and the specificity of ligand recognition. The results revealed that the aptamer of the 3′,3′-cGAMP riboswitch in the ligand-free state has a smaller binding pocket and a relatively compact structure versus that in the 3′,3′-cGAMP-bound state. The binding of the 3′,3′-cGAMP molecule to the 3′,3′-cGAMP riboswitch induces the rotation of P1 helix through the allosteric communication from the binding sites pocket containing the J1/2, J1/3 and J2/3 junction to the P1 helix. Simultaneously, these simulations also revealed that the preferential binding of the 3′,3′-cGAMP riboswitch to its cognate ligand, 3′,3′-cGAMP, over its noncognate ligand, c-di-GMP and c-di-AMP. The J1/2 junction in the 3′,3′-cGAMP riboswitch contributing to the specificity of ligand recognition have also been found.

## 1. Introduction

Riboswitch, located at the 5′ untranslated region of mRNAs, are the gene regulatory elements, which can control gene expression induced by small-molecule ligand binding [1,2,3,4,5,6]. So far, more than 20 riboswitch families have been found, due to different types of small-molecule ligands [5,7,8,9,10,11,12,13,14,15,16,17,18,19,20,21,22,23,24,25,26,27,28,29]. Riboswitches have been proposed as antibiotic drug targets [30], which have attracted structural biologists and biophysicists great attention.

**Figure 1:**
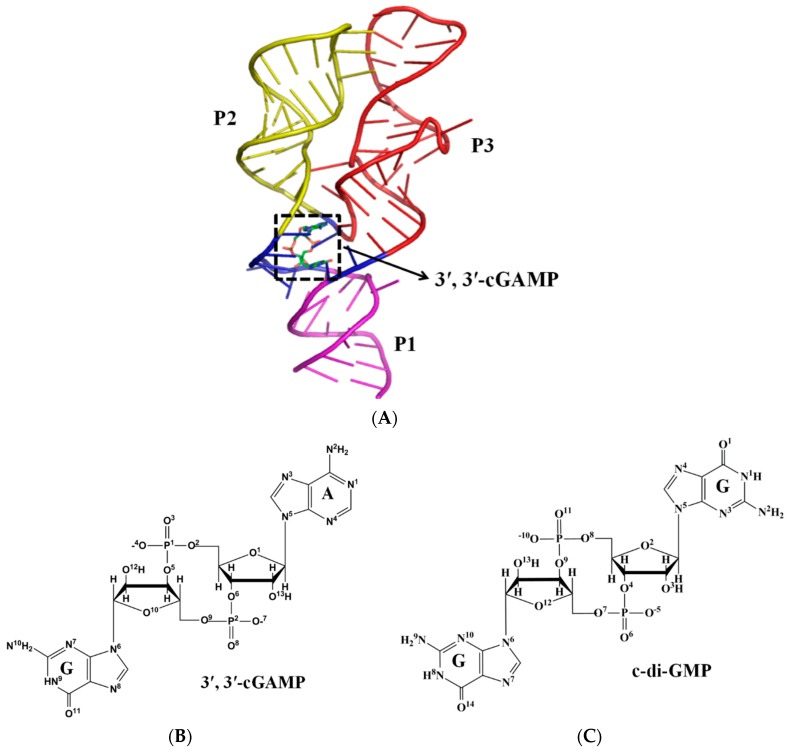
(**A**) Structure of the 3′,3′-cGAMP riboswitch in the 3′,3′-cGAMP-bound state. Structural elements are colored magenta (P1), yellow (P2), red (P3), blue (the J1/2, J2/3 and J1/3 junctions), respectively. The dotted box indicates the ligand location. Chemical formula of (**B**) the 3′,3′-cGAMP molecule and (**C**) the c-di-GMP molecule.

All known riboswitches consist of two domains, an aptamer and an expression platform [5,31]. The small-molecule ligands binding to the aptamer domain cause the structural changes in the aptamer domain and expression platform to control gene expression. In recent years, second messengers, such as c-di-GMP, c-di-AMP and 3′,3′-cGAMP, can be used as the small-molecule ligands to selectively sensing riboswitches [28,32,33,34,35,36,37,38,39]. The crystal structures of the aptamer domain of the 3′,3′-cGAMP riboswitch in the 3′,3′-cGAMP (the cognate ligand) and c-di-GMP (the noncognate ligand) bound states have been reported recently [39] (Figure 1A–C), which is similar to the aptamer domain of class I GEMM riboswitch in the c-di-GMP-bound state [34,35,40]. The structure of the 3′,3′-cGAMP riboswitch in the 3′,3′-cGAMP bound state comprises a three-helix domain (P1: G1-G8 and C76-G84, P2: C15-G40, P3: A42-U73) and a three-way helical junction (J1/2: A9-A14, J2/3: A41, J1/3: G74-C75). The three-helix domain form a tuning-fork, where the P1 helix serves as the base, and the P2, P3 helices act as the prongs. The cognate 3′,3′-cGAMP ligand locates at the three-way helical junction. The structural changes of the 3′,3′-cGAMP riboswitch have been detected in the 3′,3′-cGAMP and c-di-GMP bound states, i.e., binding of the 3′,3′-cGAMP riboswitch to the c-di-GMP molecule leads to the different orientation P2, P3 and P1 helices versus to the 3′,3′-cGAMP molecule [39]. In addition, the biochemical experiments revealed that the 3′,3′-cGAMP riboswitch binds preferentially to its cognate ligand, the 3′,3′-cGAMP molecule, over its noncognate ligand, the c-di-GMP and the c-di-AMP molecules, i.e., the specificity of ligand recognition for the 3′,3′-cGAMP riboswitch [39]. However, the exact reason of the specificity of ligand recognition for the 3′,3′-cGAMP riboswitch have not yet been detailed at the atomistic level. At the same time, it is difficult to experimentally determine the structure of the 3′,3′-cGAMP riboswitch in the ligand-free state (i.e., apo 3′,3′-cGAMP riboswitch), leaving open the question of how the apo 3′,3′-cGAMP riboswitch undergoes structural changes.

Computational techniques, and especially molecular dynamics (MD) simulations, play an important role in investigating structure and dynamics of macromelecules at the atomistic level [41,42,43,44]. MD simulations have been carried on the adenine riboswitch [45,46], the SAM-I, SAM-II riboswitches [47,48,49,50,51,52,53], the Glms riboswitch [54,55], the preQ1 riboswitch [56,57,58,59,60,61,62], the guanine riboswitch [63,64], and the c-di-GMP class I GEMM riboswitch studied in our team [65], which are useful for providing interpretations for existing experimental results and producing predictions for novel experiments on these riboswitches [66]. However, the computational investigation on the 3′,3′-cGAMP riboswitch are very limited so far.

To get a better understanding of the conformational change for the 3′,3′-cGAMP riboswitch induced by the cognate and noncognate ligands binding, molecular dynamics simulations were employed on the apo 3′,3′-cGAMP riboswitch, the 3′,3′-cGAMP riboswitch bound by the 3′,3′-cGAMP molecule and by the c-di-GMP and c-di-AMP molecules. Our main goals were (1) to address the apo 3′,3′-cGAMP riboswitch structural feature at the nucleotide level; (2) to explain specificity of ligand recognition of the 3′,3′-cGAMP riboswitch.

## 2. Results

**Figure 2:**
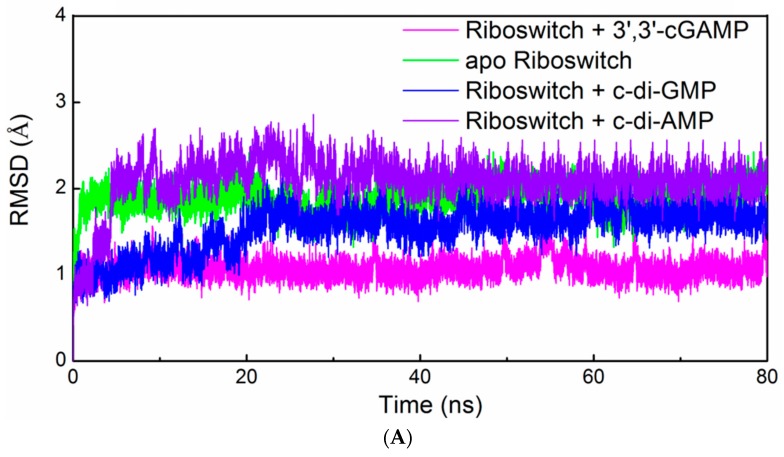

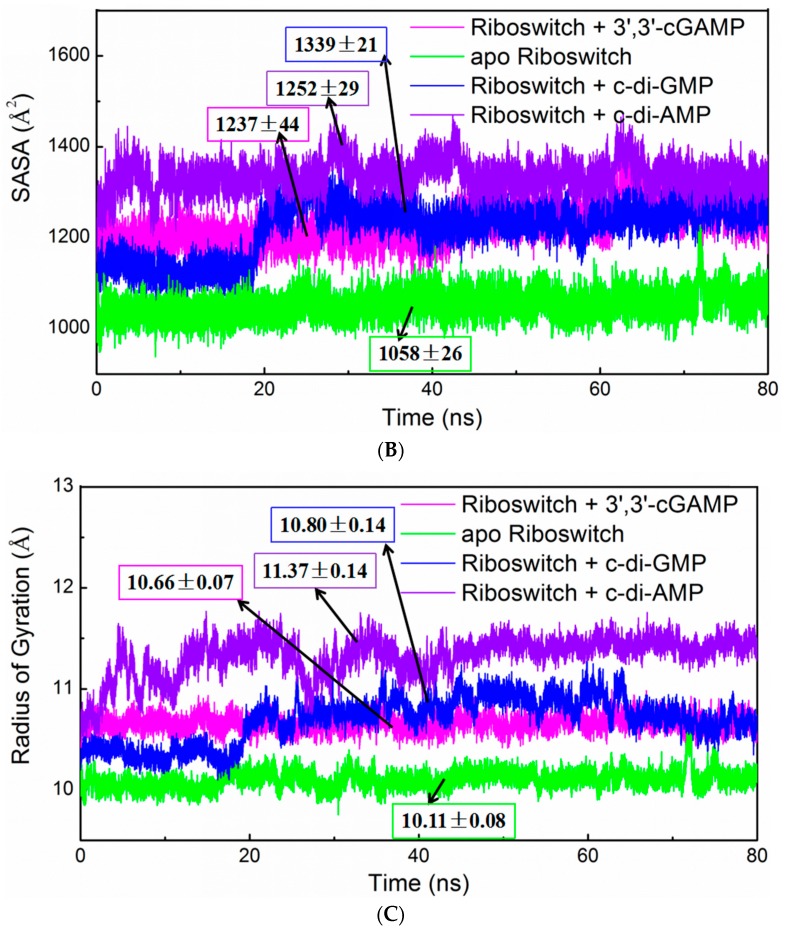
(**A**) Root-mean-square deviation (RMSD) value, (**B**) solvent accessible surface area and (**C**) radius of gyration variations of all backbone atoms in the binding pocket of the 3′,3′-cGAMP riboswitch with respect to the corresponding starting structure for the simulations of the apo riboswitch (green), riboswitch + 3′,3′-cGAMP (magentas) riboswitch + c-di-GMP (blue) and riboswitch + c-di-AMP (violet) models.

We monitored the root-mean-square deviation (RMSD) values with respect to the average structure for the apo riboswitch, riboswitch + 3′,3′-cGAMP, riboswitch + c-di-GMP, riboswitch + c-di-AMP and G20A-GEMM-I + 3′,3′-cGAMP systems (Figure S1). The small RMSD fluctuations of one simulation indicate that the system has attained equilibrium. It can be seen that the five systems had the small fluctuations of RMSD values after 20 ns, which indicate that all the studied systems have reached equilibrium after 20 ns, and the energies of these systems were also stable during the equilibrated simulation. The nucleotides that have atoms within 5 Å of 3′,3′-cGAMP (or c-di-GMP) in the riboswitch + 3′,3′-cGAMP model (or the riboswitch + c-di-GMP model) were defined as the binding pocket. The RMSD values, solvent accessible surface area (SASA) and radius of gyration (Rg) of the binding pocket for the apo riboswitch, riboswitch + 3′,3′-cGAMP, riboswitch + c-di-GMP and riboswitch + c-di-AMP systems are measured (Figure 2A–C). The RMSD values of the binding pocket in the riboswitch + 3′,3′-cGAMP model are relatively stable throughout the trajectory. The increase of the RMSD values of the binding pocket in the riboswitch + c-di-GMP, riboswitch + c-di-AMP and apo riboswitch systems predict the binding pocket has a conformational change occurring. The SASA and Rg values of binding pockets in these four systems verify the occurrence of the conformational changes, which indicate that the binding pocket in the apo riboswitch has a smaller binding pocket compare to that in the ligand-bound state.

### 2.1. Energy Calculations Revealed the Preferential Binding of the 3′,3′-cGAMP Riboswitch to 3′,3′-cGAMP over c-di-GMP and c-di-AMP

To compare the competitive binding characteristics of the 3′,3′-cGAMP riboswitch to the cognate ligand, the 3′,3′-cGAMP molecule, and to the noncognate ligand, the c-di-GMP and c-di-AMP molecules, MM-GBSA methods were used to calculate the binding free energies for the riboswitch + 3′,3′-cGAMP, riboswitch + c-di-GMP and riboswitch + c-di-AMP systems (Table 1). The binding of the 3′,3′-cGAMP riboswitch to the 3′,3′-cGAMP molecule with the binding free energy of −11.62 kcal/mol in the riboswitch + 3′,3′-cGAMP system is more energetically favorable over the binding of the 3′,3′-cGAMP riboswitch to the c-di-GMP molecule with that of −8.74 kcal/mol, and the binding of the 3′,3′-cGAMP riboswitch to the c-di-AMP molecule with the binding free energy of 13.31 kcal/mol in the riboswitch + c-di-AMP system indicate the c-di-AMP molecule can’t bind to the 3′,3′-cGAMP riboswitch, which agrees with the experiment result [39]. The ranking of binding energy predicts that the 3′,3′-cGAMP riboswitch binds preferentially to its cognate ligand, the 3′,3′-cGAMP molecule, over its noncognate ligand, the c-di-GMP and c-di-AMP molecules. In addition, the binding of the 3′,3′-cGAMP molecule to the class I GEMM riboswitch with G20A mutation with the binding free energy of −7.64 kcal/mol, which agrees with experiment result analyzed by the dissociation constants K_d_ [39] (Table S1).

**Figure 3:**
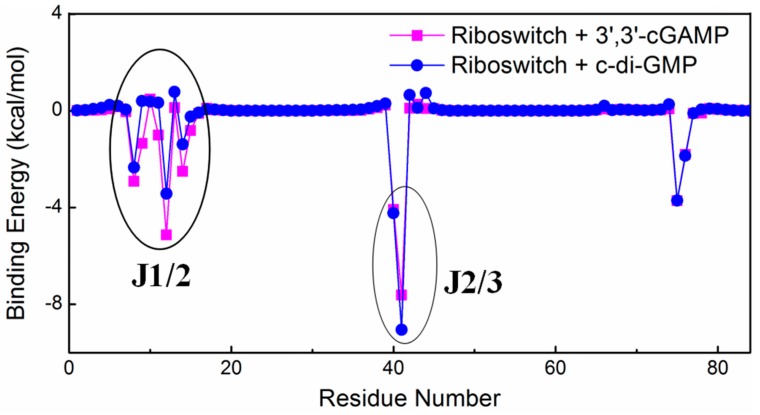
MM-GBSA energy decompositions representing the 3′,3′-cGAMP riboswitch between the 3′,3′-cGAMP riboswitch and the 3′,3′-cGAMP molecule in the riboswitch + 3′,3′-cGAMP (magentas) model, and between the 3′,3′-cGAMP riboswitch and the c-di-GMP molecule in the riboswitch + c-di-GMP (blue) model.

Furthermore, in order to address the reason of the preferential binding of the 3′,3′-cGAMP riboswitch to the 3′,3′-cGAMP molecule, the per-nucleotide free energy decompositions for the 3′,3′-cGAMP riboswitch in the riboswitch + 3′,3′-cGAMP and riboswitch + c-di-GMP systems were performed (Figure 3). The energy differences of the 3′,3′-cGAMP riboswitch mainly occur at the nucleotides around the J1/2 and J2/3 junctions, namely, the values of binding free energy decompositions at the nucleotides G8-C15 around the J1/2 junctions, G40-A42 around the J2/3 junctions are −13.10, −11.60 kcal/mol in the riboswitch + 3′,3′-cGAMP system and −5.49, −12.63 kcal/mol in the riboswitch + c-di-GMP system, respectively (Figure 3). Though the binding energy has a slight increase around the J2/3 junction, the increasing value has no influence on the ranking of binding energy. The total result of binding decompositions of the J1/2 and J2/3 junctions with −24.70 kcal/mol in the riboswitch + 3′,3′-cGAMP system is larger than that with −18.12 kcal/mol in the riboswitch + c-di-GMP system, which could explain the stronger interactions of the 3′,3′-cGAMP riboswitch with the 3′,3′-cGAMP molecule over with that of the c-di-GMP molecule.

### 2.2. Interaction Analysis between the 3′,3′-cGAMP Riboswitch and Ligands

To address the interactions of the 3′,3′-cGAMP riboswitch with the cognate ligand (i.e., the 3′,3′-cGAMP molecule) and with the noncognate liand (i.e., the c-di-GMP molecule), the percentages of all possible hydrogen bonds between the 3′,3′-cGAMP riboswitch and the 3′,3′-cGAMP molecule for the riboswitch + 3′,3′-cGAMP system, and between the 3′,3′-cGAMP riboswitch and the c-di-GMP molecule for the riboswitch + c-di-GMP system were analyzed from the equilibrated MD simulations, with the error bar analyzed by the block averaging [67,68] (Table 2). The hydrogen bond interaction was defined as follow: The distance between donor and acceptor was shorter than 3.5 Å, and the angle among donor, proton and acceptor was greater than 120° [69,70]. The are some varied hydrogen bonds between the 3′,3′-cGAMP riboswitch and the 3′,3′-cGAMP molecule for the riboswitch + 3′,3′-cGAMP model, and between the 3′,3′-cGAMP riboswitch and the c-di-GMP molecule for the riboswitch + c-di-GMP model, namely, the nucleotides G8-C15 around the J1/2 junction and G40-A42 in the J2/3 junction (Table 2). The hydrogen bond occupancies between nucleotides G8-C15 around the J1/2 junction of the 3′,3′-cGAMP riboswitch and the 3′,3′-cGAMP molecule increased 316.58%, compared to those between the 3′,3′-cGAMP riboswitch and the c-di-GMP molecule in the riboswitch + c-di-GMP model, which might cause the stronger interaction of the 3′,3′-cGAMP riboswitch with the 3′,3′-cGAMP molecule. At the same time, the occupancies of hydrogen bond between nucleotide G40-A42 in the J2/3 junction of the 3′,3′-cGAMP riboswitch and the 3′,3′-cGAMP molecule in the riboswitch + 3′,3′-cGAMP model were reduced by 79.08% compared to those between the 3′,3′-cGAMP riboswitch and the c-di-GMP molecule. Overall, however, the hydrogen bond occupancies have an increase in the riboswitch + 3′,3′-cGAMP model, which suggest the stronger interaction of the 3′,3′-cGAMP riboswitch with the 3′,3′-cGAMP molecule compare with the c-di-GMP molecule, which is in good agreement with the binding energy analysis discussed above.

**Figure 4:**
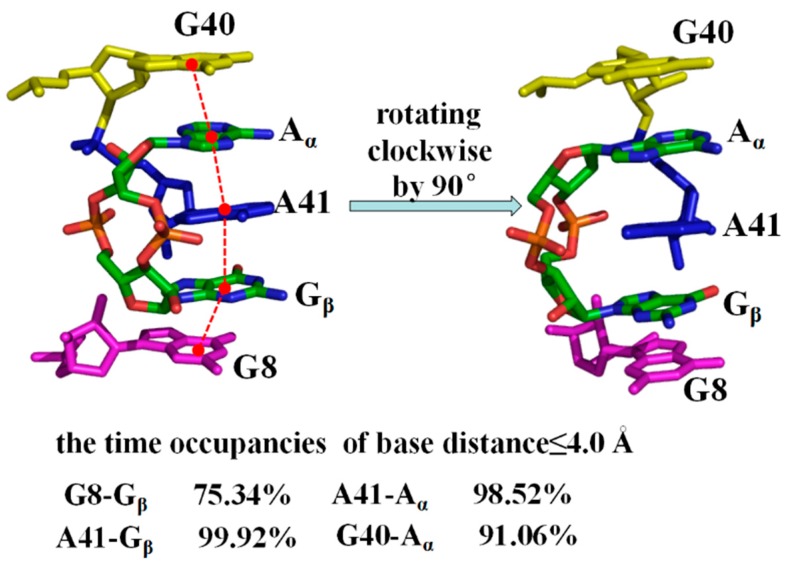
The base-stacking interactions in the binding pocket from front view and side view for the average structure of riboswitch + 3′,3′-cGAMP system. The red points indicate the centroid of the bases of G40, A_α_, A41, G_β_, G8, and the red dotted lines indicate the corresponding base-base distance.

In addition to hydrogen bond interaction, the base-stacking interactions between A_α_, G_β_ of the 3′,3′-cGAMP molecule and the 3′,3′-cGAMP riboswitch in the riboswitch + 3′,3′-cGAMP model, and between G_α_, G_β_ of the c-di-GMP molecule and the 3′,3′-cGAMP riboswitch in the riboswitch + c-di-GMP model have been detected (Figure 4 and Figure S2). The criteria for the base-stacking was defined as follow: The base-base distance ≤4.0 Å, the base-base angle ≤ 30° and overlap with each other [71]. As shown in Figure 4 and Figure S2, the base of G40, A_α_, base of A41, G_β_, base of G8 in the riboswitch + 3′,3′-cGAMP model and the base of G40, G_α_, base of A41, G_β_, base of G8 in the riboswitch + c-di-GMP model are plane-parallel approximation and overlap between the bases, which meet the angle criteria for base stacking. The percentage of distance ≤4.0 Å between base of G8 and G_β_ of 3′,3′-cGAMP, base of A41 and A_α_ of 3′,3′-cGAMP increased 45.54%, 30.58% compared to that between base of G8 and G_β_ of c-di-GMP, A41 and G_α_ of c-di-GMP, which also suggest the stronger interaction of the 3′,3′-cGAMP riboswitch with the 3′,3′-cGAMP molecule compare with the c-di-GMP molecule.

Additionally, all possible hydrogen bond interaction sites of the 3′,3′-cGAMP riboswitch in the riboswitch + 3′,3′-cGAMP system occurred at the nucleotides G8, A9, A11, A12, A14, C15 around the J1/2 junction, G40, A41, A42 around the J2/3 junction and C75 of the J1/3 junction (Table 2), which are in good agreement with energy decomposition discussed above [39]. Such hydrogen bond and base-stacking interactions bridge the P1, P2 and P3 through the J1/2, J2/3 and J1/3 junctions, which may be necessary for the orientation of the P1, P2 and P3 helices.

### 2.3. Structural Characteristics of the 3′,3′-cGAMP Riboswitch in the 3′,3′-cGAMP-Bound and c-di-GMP-Bound State

**Figure 5:**
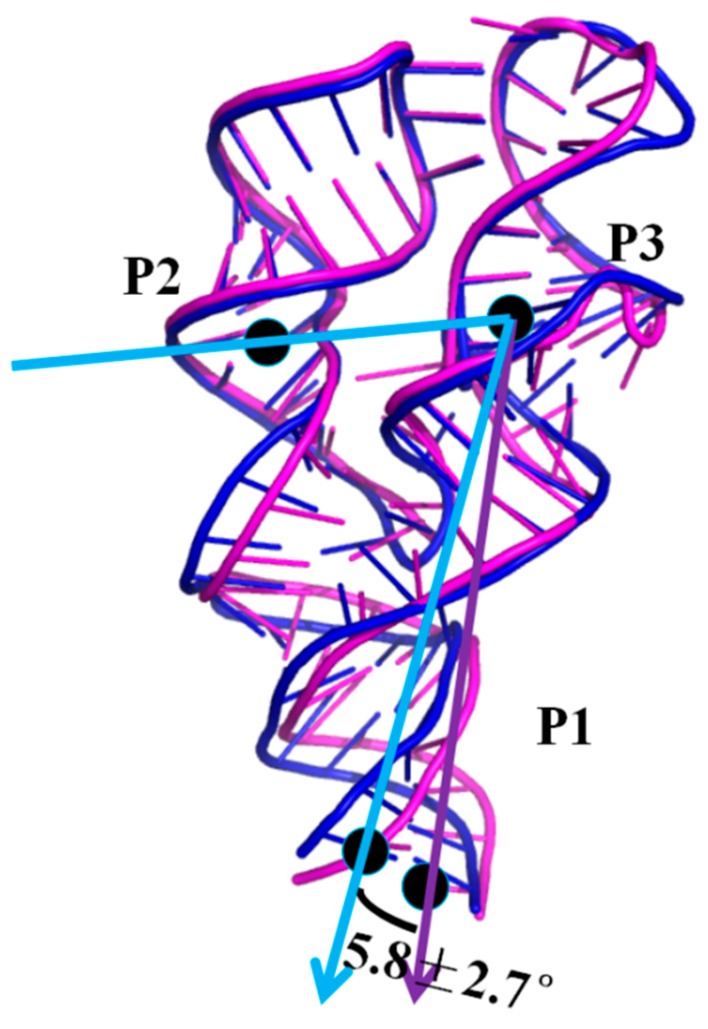
The visual superposition for the average structures of the 3′,3′-cGAMP riboswitch for riboswitch + 3′,3′-cGAMP (magentas) and riboswitch + c-di-GMP (blue) models. The angle of the P2, P3 and P1 helix are labeled for the riboswitch + 3′,3′-cGAMP (purple arrow) and riboswitch + c-di-GMP (blue arrow) models.

To address the structural change of the 3′,3′-cGAMP riboswitch in the 3′,3′-cGAMP-bound and c-di-GMP-bound state, the average structures from the equilibrated simulations of the riboswitch + 3′,3′-cGAMP and riboswitch + c-di-GMP systems are shown in Figure 5. Binding of the 3′,3′-cGAMP riboswitch to the c-di-GMP molecule leads to the rotation of P1 helix with 5.8° versus that to the 3′,3′-cGAMP molecule (Figure 5). In other words, binding the c-di-GMP molecule to the 3′,3′-cGAMP riboswitch induces a different orientation of the P2 and P3 helices versus to binding of the 3′,3′-cGAMP molecule, which is consistent with the experimental result (Figure S3) [39]. The rotation of P1 helix in the riboswitch + c-di-GMP system may be because of the differences of hydrogen bonds and base stacking interactions compared with the riboswitch + 3′,3′-cGAMP system.

**Figure 6:**
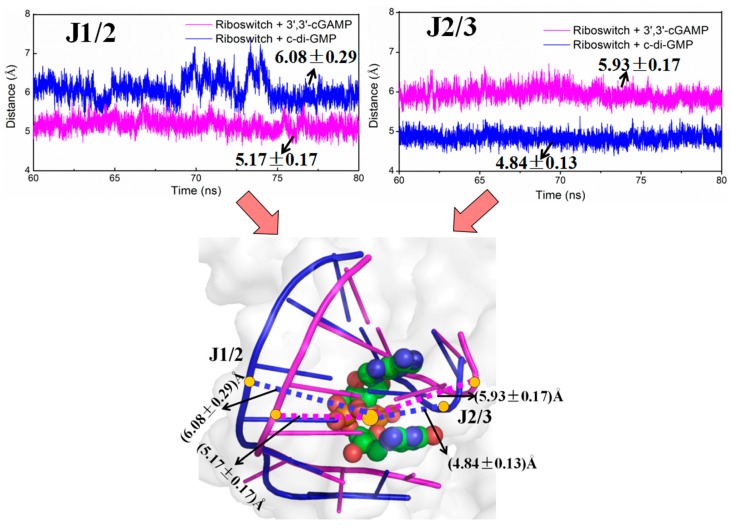
Time-dependences of distances between the J1/2 junction and ligand, between the J2/3 junction and ligand of the 3′,3′-cGAMP riboswitch for the riboswitch + 3′,3′-cGAMP (magentas) and riboswitch + c-di-GMP (blue) models. The bottom panel highlights these distance differences between the J1/2 junction and ligand, between the J2/3 junction and ligand for the riboswitch + 3′,3′-cGAMP (magentas dotted line) and riboswitch + c-di-GMP (blue dotted line) models in the two models. The yellow circles indicate the centroid of J1/2, J2/3 and ligand for the two models.

To investigate the differences of interaction of the 3′,3′-cGAMP riboswitch in the 3′,3′-cGAMP-bound and c-di-GMP-bound state, the distances between the J1/2 junction and 3′,3′-cGAMP or c-di-GMP, between the J2/3 junction and 3′,3′-cGAMP or c-di-GMP for the riboswitch + 3′,3′-cGAMP and the riboswitch + c-di-GMP systems have been measured (Figure 6). The average distance between the J1/2 junction and the 3′,3′-cGAMP molecule for the riboswitch + 3′,3′-cGAMP model is 5.17 Å, while the average distance between the J1/2 junction and the c-di-GMP molecule for the riboswitch + c-di-GMP model is 6.08 Å (Figure 6). This distance with a decrease in the riboswitch + 3′,3′-cGAMP system indicates that the J1/2 junction move toward the 3′,3′-cGAMP molecule, which result in the increase of the hydrogen bond occupancies of the J1/2 junction discussed above. As shown in Figure 6, the average distance between the J2/3 junction and the 3′,3′-cGAMP molecule for the riboswitch + 3′,3′-cGAMP model is 5.93 Å, while the average distance between the J2/3 junction and the c-di-GMP molecule for the riboswitch + c-di-GMP model is 4.84 Å. This distance with an increase in the riboswitch + 3′,3′-cGAMP system indicates that the J2/3 move away to the 3′,3′-cGAMP molecule, which result in the decrease of the hydrogen bond occupancies of the J2/3 junction and have no influence on the ranking of binding energy discussed above.

In addition, the distances between the J1/2 junction and c-di-AMP, between the J2/3 junction and c-di-AMP, between the J1/3 junction and c-di-AMP for the riboswitch + c-di-AMP system, and between the J1/3 junction and 3′,3′-cGAMP in the riboswitch + 3′,3′-cGAMP system have also been measured (Figure S4). It can be seen that the distances between J1/2, J2/3 and J1/3 junctions and c-di-AMP in the riboswitch + c-di-AMP system are larger than that in the riboswitch + 3′,3′-cGAMP system, which indicate that the J1/2, J2/3 and J1/3 junctions move away to the ligand in the riboswitch + c-di-AMP system. The movements of the J1/2, J2/3 and J1/3 junctions may result in the unbinding of the c-di-AMP molecule to the 3′,3′-cGAMP riboswitch, which is consistent with the binding energy discussed above.

### 2.4. Structural Characteristics of the 3′,3′-cGAMP Riboswitch in the Ligand-Free and 3′,3′-cGAMP-Bound State

**Figure 7:**
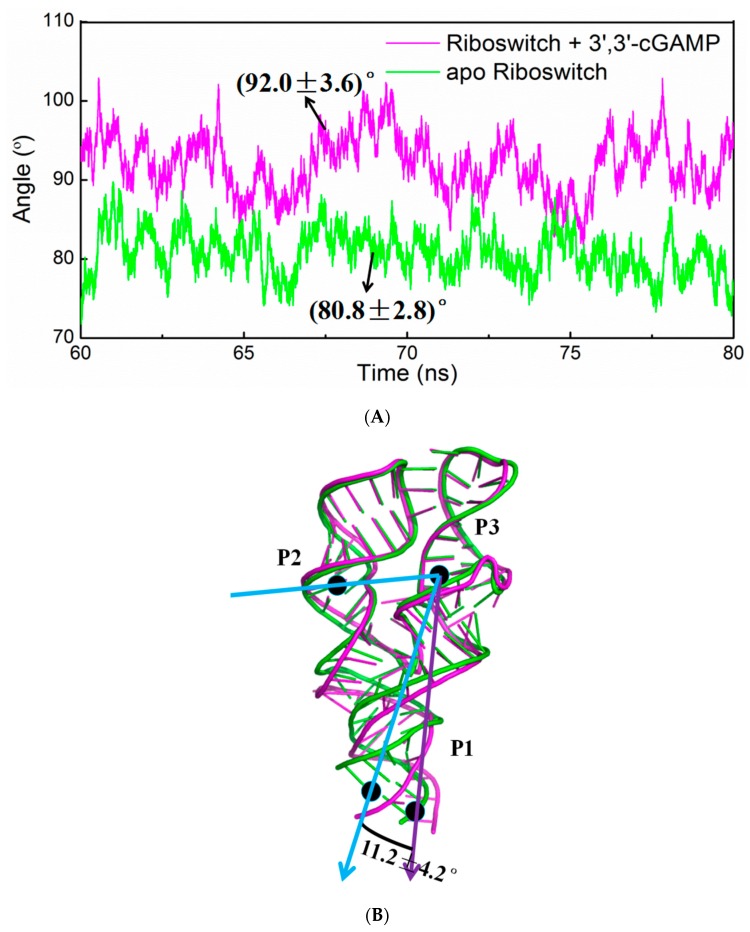
(**A**) Time-dependences of rotation angle of P2, P3 and P1 helix of the 3′,3′-cGAMP riboswitch for riboswitch + 3′,3′-cGAMP (magentas) and apo riboswitch (green) models; (**B**) the visual superposition for the average structures of the 3′,3′-cGAMP riboswitch for riboswitch + 3′,3′-cGAMP (magentas) and apo riboswitch (green) models. The angle of the P2, P3 and P1 helix are labeled for the riboswitch + 3′,3′-cGAMP (purple arrow) and apo riboswitch (blue arrow) models.

**Figure 8:**
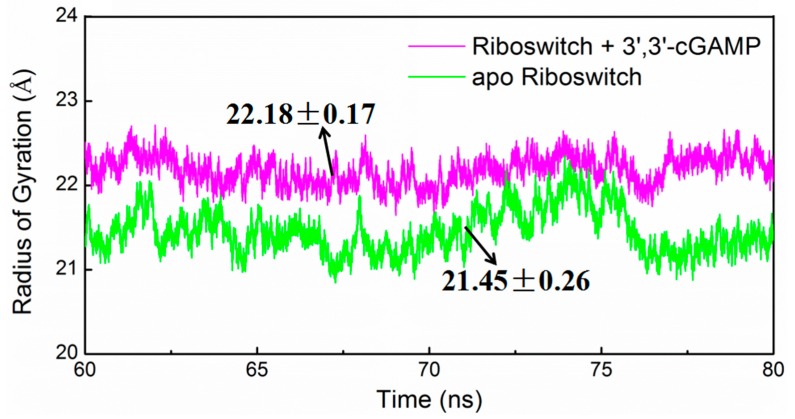
The radius of gyration value variations of the backbone atoms of the 3′,3′-cGAMP riboswitch for the apo riboswitch (green) and riboswitch + 3′,3′-cGAMP (magentas) models.

To address the structural change of the 3′,3′-cGAMP riboswitch in the ligand-free and 3′,3′-cGAMP-bound state, the angle changes among the centroid of P2, P3 and P1 of 3′,3′-cGAMP riboswitch of the apo riboswitch and riboswitch + 3′,3′-cGAMP systems have been analyzed and are shown in Figure 7A, and the average structures are shown in Figure 7B. It can be seen that the average angle among the centriod of P2, P3 and P1 decrease from 92.0° in the riboswitch + 3′,3′-cGAMP system to 80.8° in the apo riboswitch system, which indicates that the 3′,3′-cGAMP riboswitch leads to the rotation of P1 helix with 11.2° induced by the 3′,3′-cGAMP molecule unbinding (Figure 7A,B). Such rotation results in the tighter packing of riboswitch in its free state that is validated by the radius of gyration (Rg) analysis of the 3′,3′-cGAMP riboswitch in the apo riboswitch and riboswitch + 3′,3′-cGAMP systems (Figure 8). The Rg of the apo riboswitch system was lower than that of the riboswitch + 3′,3′-cGAMP system (Figure 8). The P1 helix rotation in the apo riboswitch system may be because of the destruction of hydrogen bonds and base stacking interactions compared with the riboswitch + 3′,3′-cGAMP system. The destruction of these interactions also disturbs the 3′,3′-cGAMP binding pocket conformation.

**Figure 9:**
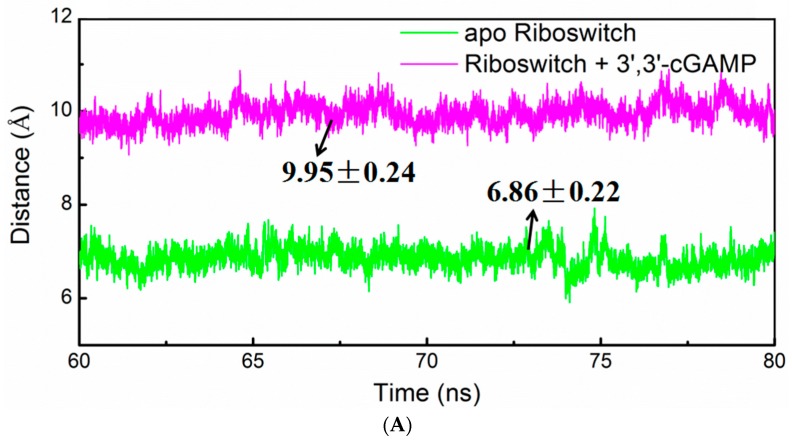

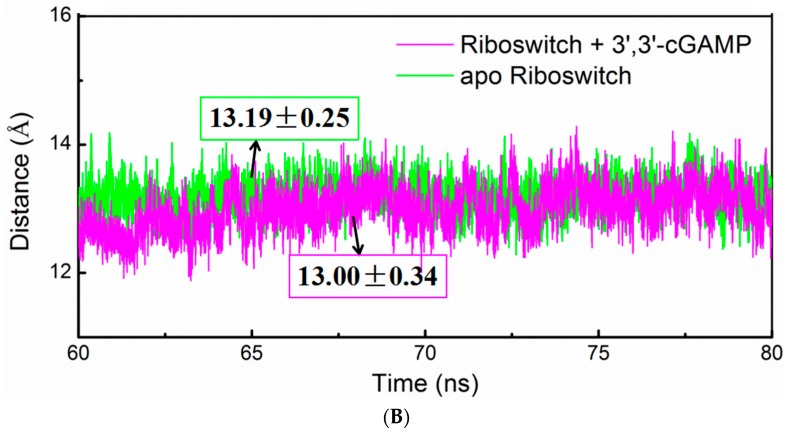
Time-dependences of distances (**A**) between J1/2 and J2/3 junctions, and (**B**) between J1/2 and J1/3 junctions of riboswitch for the apo riboswitch (green) and riboswitch + 3′,3′-cGAMP (magentas) models.

To investigate the binding pocket conformational change of the 3′,3′-cGAMP riboswitch in the apo riboswitch and riboswitch + 3′,3′-cGAMP systems, the distances between J1/2 and J2/3, and between J1/2 and J1/3 in the riboswitch over the simulation times for the apo riboswitch and riboswitch + 3′,3′-cGAMP models has been also analyzed (Figure 9A,B). The average distance between the J1/2 junction and the J2/3 junction decreases from 9.95 Å in the riboswitch + 3′,3′-cGAMP system to 6.86 Å in the apo riboswitch system (Figure 9A). At the same time, the average distance between the J1/2 junction and the J1/3 junction is nearly same in two models (Figure 9B). This distance with a decrease indicates that the J1/2 junction and the J2/3 junction of the 3′,3′-cGAMP riboswitch move close to each other, which may favor the smaller pocket in the apo riboswitch system and rotation of P1 helix.

### 2.5. Principal Component Analysis of the 3′,3′-cGAMP Riboswitch in the Ligand-Free and 3′,3′-cGAMP-Bound State

Principal component analysis was used to analyze the trajectories from the corresponding simulations to examine the dominant 3′,3′-cGAMP riboswitch dynamic motions in the apo riboswitch and riboswitch + 3′,3′-cGAMP models (Table 3). The first 3 principal components, PC1-PC3, account for about 90% of all the motion modes. In the riboswitch + 3′,3′-cGAMP system, the first three principal modes clearly observed a bending motion between P3 and P1 helices, which may cause the closing and opening of P1 helix along the vertical plane and have no obvious rotation of P1 helix on the horizontal plane (Movie S1, S2 and S3). However, in the apo riboswitch system, the first three principal modes obviously showed a twist motion between P3 and P1 helix (Movie S4, S5 and S6), which may induce the rotation of P1 helix on the horizontal plane. Average structure analyses of the 3′,3′-cGAMP riboswitch support the PCA dominant motions (Figure 7).

### 2.6. Allosteric Communication of the 3′,3′-cGAMP Riboswitch from Binding Sites Pocket to the P1 Helix

**Figure 10:**
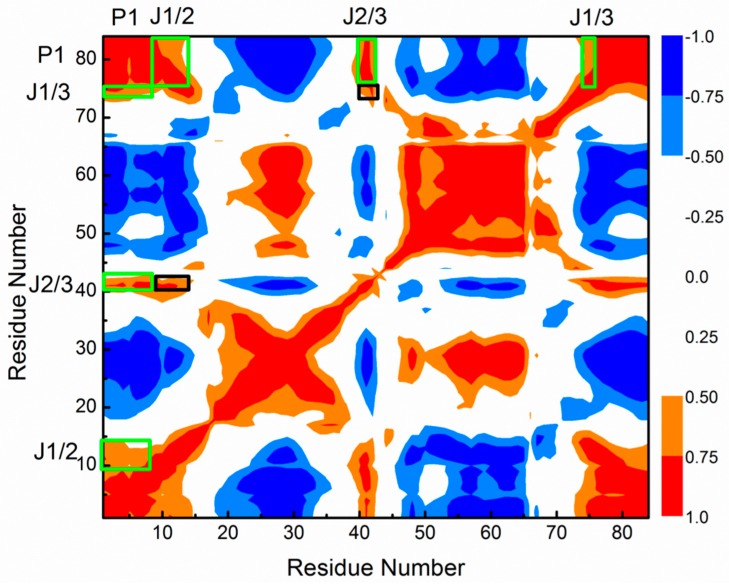
Dynamical cross-correlation map calculating from the first 10 ns of the apo riboswitch model with the specific sub-regions squared in green and black. These key subregions squared in green and black involve in the direct and indirect allosteric communication networks, respectively.

To explore the allosteric communications, dynamic cross-correlation map (DCCM) of the allosteric process of 3′,3′-cGAMP riboswitch induced by the 3′,3′-cGAMP molecule have been analyzed (Figure 10). The correlation values vary from −1 (high anticorrelation, blue) to 1 (high correlations, red). Because the 3′,3′-cGAMP molecule binding sites mainly occurred at the nucleotides G8, A9, A11, A12, A14, C15 around the J1/2 junction, G40, A41, A42 around the J2/3 junction, C75 of the J1/3 junction, the DCCM also appeared in those regions of the 3′,3′-cGAMP riboswitch. It can be found from Figure 10 that the large correlated motions of the J2/3 junction vs the J1/2 and J1/3 junctions occur obviously with large correlated motions of the J1/2 and J1/3 junctions vs the P1 helix, which predict the indirect allosteric communication between the J2/3 junction and the P1 helix via the J1/2 and J1/3 junctions, and direct allosteric communication between the J1/2 and J1/3 junctions and the P1 helix.

Simultaneously, the DCCMs of the 3′,3′-cGAMP riboswitch for equilibrated simulation of the riboswitch + 3′,3′-cGAMP, riboswitch + c-di-GMP and apo riboswitch systems are shown in Figure S5A–C. It can be seen from Figure 10 and Figure S5A,B that the DCCMs of the 3′,3′-cGAMP riboswitch extracted from the equilibrated simulation of the riboswitch + 3′,3′-cGAMP, riboswitch + c-di-GMP systems show very similar features with that from the allosteric process induced by the 3′,3′-cGAMP molecule, which indicate that the similar allosteric communication networks discussed above also exist in the equilibrated riboswitch + 3′,3′-cGAMP and riboswitch + c-di-GMP systems. Comparison of the DCCM of the equilibrated simulations of the apo riboswitch (Figure S5C) system with that of the riboswitch + 3′,3′-cGAMP (Figure S5A) system show distinct features. Namely, in the case of riboswitch + 3′,3′-cGAMP simulation (Figure S5A), a large correlated motion is observed, in contrast, in the apo riboswitch system, the correlated motions have diminished, which indicate that the allosteric communication network from the 3′,3′-cGAMP binding sites to P1 helix discussed above was reduced or abolished in the apo riboswitch system (Figure S5C). Overall, the allosteric communication between the 3′,3′-cGAMP binding pocket and the P1 helix can obviously occur induced by the ligand binding, such as the 3′,3′-cGAMP molecule or the c-di-GMP molecule.

## 3. Discussion

### Allosteric Communication Network from 3′,3′-cGAMP Binding Sites to P1 Helix

As discussed, the indirect and direct allosteric communication occurred at the 3′,3′-cGAMP riboswitch induced by the 3′,3′-cGAMP molecule binding, which relates to the structural changes/interactions around the J1/2, J2/3, J1/3 junctions and P1 helix. Namely, the 3′,3′-cGAMP molecule binding to the 3′,3′-cGAMP riboswitch causes the movement of the J1/2, J2/3 and J1/3 junctions and the larger binding pocket, which favors the combination of the 3′,3′-cGAMP riboswitch with the 3′,3′-cGAMP molecule. Such movement of the J1/2, J2/3 and J1/3 junctions relates to the formation of base-stacking and hydrogen bond interactions between the nucleotides G8, A9, A11, A12, A14, C15 around the J1/2 junction, G40, A41, A42 around the J2/3 junction, C75 of the J1/3 junction and the 3′,3′-cGAMP molecule (Table 1 and Figure 4), which suggest that the motions of the J2/3 junction correlated with the movement of the J1/2 and J1/3 junctions. Furthermore, the J1/2, J1/3 junctions are directly linked with the P1 helix, which indicate that the movement of the J1/2, J1/3 junctions correlated to the rotation with 11.2°of the P1 helix.

When the binding of the 3′,3′-cGAMP riboswitch to the noncognate ligand, the c-di-GMP molecule, the same indirect allosteric and direct allosteric communication networks have been detected versus to the cognate ligand, the 3′,3′-cGAMP molecule. However, the structural change and interaction differences of binding pocket have been found between the riboswitch + 3′,3′-cGAMP and riboswitch + c-di-GMP systems. When the c-di-GMP molecule binding to the 3′,3′-cGAMP riboswitch causes the movement of the J1/2 junction away from the c-di-GMP molecule and the decrease of hydrogen bonds at nucleotides G8, A9, A11, A12, A14 and C15 of J1/2 versus to the 3′,3′-cGAMP molecule, which leads to the binding energy decrease of the J1/2 junction. Though the binding energy of the J2/3 junction in the 3′,3′-cGAMP riboswitch has a slight increase, due to the structural change of the J2/3 junction when the binding of the 3′,3′-cGAMP riboswitch to the c-di-GMP molecule versus to the 3′,3′-cGAMP molecule, it does not affect the binding ranking. Finally, the results revealed that the combination of 3′,3′-cGAMP riboswitch with the 3′,3′-cGAMP molecule is energetically favorable for the combination of the 3′,3′-cGAMP riboswitch with the c-di-GMP molecule. The 3′,3′-cGAMP riboswitch binding to the c-di-GMP molecule causes the difference of structure and interactions of the binding pocket (i.e., J1/2, J2/3, J1/3) versus to the 3′,3′-cGAMP molecule. Furthermore, the correlations of the J1/2, J2/3 and J1/3 junctions to the P1 helix induced by the c-di-GMP molecule binding causes the rotation of P1 helix with 5.8° versus that by the 3′,3′-cGAMP binding.

## 4. Materials and Methods

### 4.1. Initial Structures

From previous studies, the initial structure of the 3′,3′-cGAMP riboswitch in the 3′,3′-cGAMP-bound state (assigned as riboswitch + 3′,3′-cGAMP model) was taken from the crystal structure published by Ren et al. (PDB code: 4YAZ) [39] (Figure 1). To obtain the starting structure of the apo 3′,3′-cGAMP riboswitch, the riboswitch + 3′,3′-cGAMP model was edited to strip off the 3′,3′-cGAMP molecule and was assigned as the apo riboswitch model. In order to investigate the specificity of ligand recognition, the structure of the 3′,3′-cGAMP riboswitch in the c-di-GMP-bound state (assigned as riboswitch + c-di-GMP) was constructed from the crystal structure (PDB code: 4yb0) [39]. The initial structure of the 3′,3′-cGAMP riboswitch in the c-di-AMP-bound state (assigned as riboswitch + c-di-AMP) was constructed based on the structure of the riboswitch + c-di-GMP model. The coordinates of the c-di-AMP molecule extracted from the crystal structure (PDB code: 3MUV) [39] were superimposed onto the riboswitch + c-di-GMP model as the substitute for the c-di-GMP molecule. To elucidate the 3′,3′-cGAMP molecule binding to the class I GEMM riboswitch, the initial structure of the class I GEMM riboswitch with G20A mutation in the 3′,3′-cGAMP-bound state (assigned as G20A-GEMM-I + 3′,3′-cGAMP model) was taken from the crystal structure (PDB code: 4YB1) [39]. In the five models, we retained all ions and water coordinates. Na^+^ were added to the negative position of RNA in each model to achieve electroneutrality. The systems were solvated in a rectangular box of TIP3P waters, with 10 Å padding of the solvent shell in all directions. Three MD simulations were repeated for each model to check robustness of the results although the starting structure was constructed from the X-ray crystal result, which yield a non-distinguishable result, e.g., the similar binding energy and average structure results (Table S2 and Figure S6A–C), when simulations reach equilibrium.

### 4.2. Ligand Force Field Parameter

The coordinates of the 3′,3′-cGAMP and c-di-GMP ligand were extracted from riboswitch + 3′,3′-cGAMP and riboswitch + c-di-GMP models, respectively, and the ligand structure was optimized and RESP [72] charges were calculated at the HF/6-31G* level of theory using the Gaussian09 program [73]. Generalized Amber force field parameters [74] of the 3′,3′-cGAMP ligand were generated using the Antechamber program in the Amber.

### 4.3. Molecular Dynamics Simulation Protocols

All energy minimization and MD simulations were performed using the sander module in AMBER16 package [75] with the parm99 [76,77] and parmbsc0 force field [78] for RNA nucleotide. MD simulation in the NPT ensemble at the constant pressure and temperature of 1 bar and 300 K was carried out for each system. For each simulation, a 2 fs integration step was used. The MD protocols details are available in the Supplementary Materials and our previous studies [65,79,80,81].

### 4.4. Principal Component Analysis

Principal component analysis (PCA) can be used to probe the most prominent characteristic of the dynamics of the studied system. The PCA method details are available in the Supplementary Materials and our previous studies [65].

### 4.5. Free-Energy Analyses

The binding free energy was computed using the molecular mechanics Generalized Born Surface Area (MM-GBSA) method [82,83,84,85].

Δ*G*_binding_ = *G*_complex_ − *G*_ligand_ − *G*_receptor_

where Δ*G*_binding_ is the binding free energy, *G*_complex_, *G*_ligand_ and *G*_receptor_ are the free energy of complex, ligand and receptor, respectively. Computational details can be found in the Supplementary Materials and our previous studies [65,79,80].

### 4.6. Trajectory Analyses

The root-mean-square deviation (RMSD), the hydrogen interaction, the base-stacking interaction and the correlation coefficients [75,86] were calculated by Cpptraj and Ptraj in the AMBER16 program [75] in this work. Details of the correlation coefficients analyses are available in our previous studies [65,79,80,81].

## 5. Conclusions

Molecular dynamics simulations have been performed for the aptamer domain of the 3′,3′-cGAMP riboswitch in the ligand-free, the cognate ligand-bound (i.e., 3′,3′-cGAMP-bound) and the noncognate ligand-bound (i.e., c-di-GMP-bound and c-di-AMP-bound) states. The results demonstrated that in the absence of ligand, the distance between J1/2 and J2/3 with a decrease of 3.09 Å causes a smaller binding pocket containing the J1/2, J2/3 and J1/3 junctions. When the binding of the 3′,3′-cGAMP riboswitch to the 3′,3′-cGAMP molecule, the hydrogen bonds and stacking interactions between the nucleotides G8, A9, A11, A12, A14, C15 around the J1/2 junction, G40-A42 around the J2/3 junction, C75 of the J1/3 junction and the 3′,3′-cGAMP molecule increase the size of the 3′,3′-cGAMP binding site pocket. Furthermore, the structural change of the binding pocket, due to the 3′,3′-cGAMP binding causes the P1 helix rotation with 11.2° versus to the apo 3′,3′-cGAMP structure. The correlation and interaction analysis presents the direct and indirect allosteric communication networks in the 3′,3′-cGAMP riboswitch for the rotation of P1 helix. The allosteric communication network involves from the 3′,3′-cGAMP binding site pocket, i.e., J1/2, J2/3, J1/3, communicating to P1 helix. In addition, free energy calculations demonstrated that the combination of the 3′,3′-cGAMP riboswitch with the 3′,3′-cGAMP molecule is energetically favorable for the combination of the 3′,3′-cGAMP riboswitch with the c-di-GMP (or c-di-AMP) molecule. The visual analyses of the 3′,3′-cGAMP riboswitch reveal that the binding of the 3′,3′-cGAMP riboswitch to the c-di-GMP molecule induces the movement of the J1/2 junction away from the c-di-GMP versus to the 3′,3′-cGAMP molecule. Such movement of the J1/2 junction results in the decrease of hydrogen bonds at nucleotides G8, A9, A11, A12, A14 and C15 of the J1/2 junction, which leads to the binding energy decrease of the J1/2 junction as discussed in the energy decomposition analysis. As expected, the c-di-GMP molecule binding causes the P1 helix rotation with 5.8° compared with the 3′,3′-cGAMP molecule binding through the direct and indirect allosteric communication involving from the J1/2, J2/3 and J1/3 junctions to the P1 helix. These results provide the better understanding for the structural change of the 3′,3′-cGAMP riboswitch induced by ligands and the specificity of ligand recognition.

## Figures and Tables

**Table 1:** MM-GBSA free energy (kcal·mol^−1^) components for the riboswitch + 3′,3′-cGAMP and riboswitch + c-di-GMP models.

Energies	Riboswitch + 3′,3′-cGAMP	Riboswitch + c-di-GMP	Riboswitch + c-di-AMP
Receptor	3′,3′-cGAMP riboswitch	3′,3′-cGAMP riboswitch	3′,3′-cGAMP riboswitch
Ligand	3′,3′-cGAMP	c-di-GMP	c-di-AMP
Δ*E*_ele_	1349.93	2699.95	2647.28
Δ*E*_vdw_	−81.92	−78.61	−67.59
Δ*G*_np/solv_	−7.26	−7.08	−6.73
Δ*G*_pb/solv_	−1296.48	−2645.96	−2587.86
Δ*G*_np_	−89.18	−85.69	−74.32
Δ*G*_pb_	53.45	54.00	59.42
Δ*H*	−35.73	−31.69	−14.90
*T*Δ*S*	−24.11	−22.95	−28.21
Δ*G*_binding_	−11.62 (0.12) ^a^	−8.74 (0.11) ^a^	13.31 (0.12) ^a^
Δ*G*_exp_	−9.82	−8.28	---
k_d_	0.07 uM	0.93 uM	

^a^: The values in parentheses are standard errors; ---: no binding; Δ*E*_ele_: the electrostatic energy; Δ*E*_vdw_: the van der Waals energy; Δ*G*_np/solv_: the non-polar solvation free energy; Δ*G*_pb/solv_: the electrostatic solvation free energy; K_d_: the dissociation constant. Δ*G*_pb_ = Δ*E*_ele_ + Δ*G*_pb/solv_; Δ*G*_np_ = Δ*E*_vdw_ + Δ*G*_np/solv_; Δ*H* = Δ*G*_np_ + Δ*G*_pb_ + Δ*E*_int_; Δ*G*_binding_ = Δ*H* − *T*Δ*S*; Δ*G*_exp_ = RTlnk_d_/(1000 × 4.184).

**Table 2:** The occupancies (%) of hydrogen bonds with the error bars between the 3′,3′-cGAMP riboswitch and the 3′,3′-cGAMP molecule for the riboswitch + 3′,3′-cGAMP model, and between the 3′,3′-cGAMP riboswitch and the c-di-GMP molecule for the riboswitch + c-di-GMP model.

Riboswitch + 3′,3′-cGAMP			Riboswitch + c-di-GMP		
3′,3′-cGAMP Riboswitch∙∙∙3′,3′-cGAMP	Occupancies (%)	Error	3′,3′-cGAMP Riboswitch∙∙∙3′,3′-cGAMP	Occupancies (%)	Error
(G8)O2′-H∙∙∙O^9^(3′,3′-cGAMP)	55.00	3.5	---	---	---
(G8)O13-H∙∙∙O^10^(3′,3′-cGAMP)	48.44	2.1	---	---	---
(A9)O2′∙∙∙H-O^12^(3′,3′-cGAMP)	77.12	5.0	---	---	---
(A11)N6-H∙∙∙O^12^(3′,3′-cGAMP)	91.54	1.0	(A11)N6-H∙∙∙O^13^(c-di-GMP)	36.56	2.8
(A11)N7∙∙∙H-O^12^(3′,3′-cGAMP)	5.12	1.4	---	---	---
(A12)N1∙∙∙H-N^10^(3′,3′-cGAMP)	99.76	0.1	(A12)N1∙∙∙H-N^9^(c-di-GMP)	88.68	3.8
(A12)N6-H∙∙∙O^3^(3′,3′-cGAMP)	96.50	1.4	(A12)N6-H∙∙∙N^10^(c-di-GMP)	78.96	3.5
(A12)N6-H ∙∙N^7^(3′,3′-cGAMP)	43.88	1.3	(A12) N6-H∙∙∙N^9^(c-di-GMP)	30.20	4.7
(A12)N6-H∙∙∙P^1^(3′,3′-cGAMP)	5.22	0.4	---	---	---
(A14)N2-H∙∙∙N^3^(3′,3′-cGAMP)	40.22	1.2	(A14)N6-H∙∙∙O^1^(c-di-GMP)	80.28	3.0
(A14)N1∙∙∙H-N^2^(3′,3′-cGAMP)	38.36	1.3	(A14) N6-H∙∙∙N^4^(c-di-GMP)	6.48	0.8
(A14)N6∙∙∙H-N^2^(3′,3′-cGAMP)	6.80	2.6	---	---	---
(A14)N6∙∙∙H-N^2^(3′,3′-cGAMP)	6.56	2.8	---	---	---
(C15)N4-H∙∙∙N^3^(3′,3′-cGAMP)	16.70	1.4	---	---	---
(C15)N3∙∙∙H-N^2^(3′,3′-cGAMP)	6.52	2.6	---	---	---
(G40)O2P∙∙∙H-O^13^(3′,3′-cGAMP)	27.46	1.9	(G40)O2′∙∙∙H-N^2^(c-di-GMP)	58.92	2.1
(G40)P∙∙∙H-O^13^(3′,3′-cGAMP)	19.46	1.7	---	---	---
(A41)N6-H∙∙∙O^4^(3′,3′-cGAMP)	99.30	0.2	(A41)N6-H∙∙∙O^11^(c-di-GMP)	99.72	0.1
(A41)O2P∙∙∙H-O^13^(3′,3′-cGAMP)	70.34	5.4	(A41)O2P∙∙∙H-O^3^(c-di-GMP)	99.20	0.4
(A41)O2′-H∙∙∙N^8^(3′,3′-cGAMP)	16.68	5.0	(A41) O2′-H∙∙∙N^7^(c-di-GMP)	49.96	6.1
---	---	---	(A41)O5′∙∙∙H-N^2^(c-di-GMP)	5.72	3.1
(A42)N3∙∙∙H-N^2^(3′,3′-cGAMP)	22.20	1.3	(A42)O2′∙∙∙H-N^2^(c-di-GMP)	21.00	3.6
(C75)N3∙∙∙H-N^10^(3′,3′-cGAMP)	99.52	0.4	(C75)N4-H∙∙∙N^8^(c-di-GMP)	98.84	0.9
(C75)N4-H∙∙∙N^9^(3′,3′-cGAMP)	98.40	0.6	(C75)N3∙∙∙H-N^9^(c-di-GMP)	97.52	0.8
(C75)O2∙∙∙H-N^10^(3′,3′-cGAMP)	52.02	1.8	(C75)N4-H∙∙∙O^14^(c-di-GMP)	60.40	7.6
(C75)N4-H∙∙∙O^11^(3′,3′-cGAMP)	31.26	4.5	(C75)O2∙∙∙H-N^9^(c-di-GMP)	37.24	7.0

∙∙∙ is the hydrogen bond; --- indicts no hydrogen bond occurrence.

**Table 3:** Percentages of occupancy times of the first three principal components during the simulations of the apo riboswitch and riboswitch + 3′,3′-cGAMP models.

Systems	PC1	PC2	PC3	PCs ^a^
apo riboswitch	69.85	12.34	9.01	91.20
Riboswitch +3′,3′-cGAMP	72.07	11.50	7.39	90.96

^a^: PCs represents sum of PC1, PC2 and PC3.

## Supplementary Materials

The following are available online at http://www.mdpi.com/1422-0067/19/11/3527/s1, Figure S1: Root-mean-square deviation (RMSD) values of all backbone atoms of the 3′,3′-cGAMP riboswitch with respect to the corresponding average structure for the simulations of apo riboswitch (green), riboswitch + c-di-GMP (blue), riboswitch + 3′,3′-cGAMP (magentas), riboswitch + c-di-AMP (violet) and G20A-GEMM-I + 3′,3′-cGAMP (cyan) models, Figure S2: The base-stacking interactions in the binding pocket for the average structure of the riboswitch + c-di-GMP system, Figure S3: The visual superposition for the average structures of the 3′,3′-cGAMP riboswitch for riboswitch + 3′,3′-cGAMP (magentas) and riboswitch + c-di-GMP (magentas)models. The P1 helix forming the fixed domain, Figure S4: Time-dependences of distances (a) between the J1/2 junction and ligand, (b) between the J2/3 junction and ligand, (c) between the J1/3 junction and ligand of the 3′,3′-cGAMP riboswitch for the riboswitch + 3′,3′-cGAMP (magentas) and riboswitch + c-di-AMP (violet) systems, Figure S5: Dynamical cross-correlation map from the last 10 ns of the 3′,3′-cGAMP riboswitch for the (a) riboswitch + 3′,3′-cGAMP, (b) riboswitch + c-di-GMP and (c) apo riboswitch models with the specific sub-regions squared in green and black. These key subregions squared in green and black involve in the direct and indirect allosteric communication networks, respectively, Figure S6: The visual superposition for the average structures of the 3′,3′-cGAMP riboswitch for the three repeated MD simulations (replica 1—white, replica 2:—yellow, replica 3—cyan) of (a) riboswitch + 3′,3′-cGAMP, (b) riboswitch + c-di-GMP and (c) apo riboswitch models, Table S1: MM-GBSA free energy (kcal·mol-1) components for the G20A-GEMM-I + 3′,3′-cGAMP model, Table S2: MM-GBSA free energy (kcal·mol-1) components for three repeated molecular dynamics (MD) simulations (replica 1, replica 2, replica 3) of riboswitch + 3′,3′-cGAMP, riboswitch + c-di-AMP, riboswitch + c-di-GMP and G20A-GEMM-I + 3′,3′-cGAMP models, Video S1, Video S2 and Video S3: PC1, PC2 and PC3 for the riboswitch + 3′,3′-cGAMP model, Video S4, Video S5 and Video S6: PC1, PC2 and PC3 for the apo model.
