# Supplementary material for: Structural Studies of the 3′,3′-cGAMP Riboswitch Induced by Cognate and Noncognate Ligands Using Molecular Dynamics Simulation
Source: Int J Mol Sci. 2018 Nov 9;19(11):3527. doi: 10.3390/ijms19113527 (PMC6274999; doi:10.3390/ijms19113527)
Supplement: Supplementary file 1 [file ijms-19-03527-s001.zip › ijms-370704-supplementary-proofread/ijms-370704-supplementary-proofread.doc]

**Structural Studies of the 3′,3′-cGAMP Riboswitch Induced by Cognate and Noncognate Ligands Using the Molecular Dynamics Simulation**

Chaoqun Li^1^, Xiaojia Zhao^1^, Xiaomin Zhu^1^, Pengtao Xie^1^ and Guangju Chen^2^

^1^*College of Chemistry, Chemical Engineering and Materials, Handan University, No.530 North Xueyuan Road, Hanshan District, Han Dan, Hebei province, P.R.China*

^2^College of Chemistry, Beijing Normal University, 19# Xinjiekouwai Street, Beijing 100875, P. R. China

**Supplementary Materials**

**Molecular dynamics simulation protocols**

The protocol for all molecular dynamics (MD) simulations is described as follows: (1) The systems were first minimized to remove unfavorable contacts. Four rounds of minimization (5000 steps of each minimization) were carried out to hold the 3′,3′-cGAMP molecule and 3′,3′-cGAMP riboswitch with harmonic forces of 100, 75, 50, and 25 kcal·mol^-1^·Å^-2^. The fifth round of minimization with 10000 steps was performed without restraint. (2) The systems were heated up from 0 to 300 K in 120 ps with constant volume, while harmonic force of 25 kcal·mol^-1^·Å^-2^ was used to restrain the positions of the 3′,3′-cGAMP molecule and 3′,3′-cGAMP riboswitch. (3) The restraints on the 3′,3′-cGAMP molecule and 3′,3′-cGAMP riboswitch were removed and a 200-ps’ equilibration in the NPT ensemble at the constant pressure and temperature of 1 bar and 300 K, respectively, was carried out for each system. (4) Finally, an 80 ns MD simulation was carried out for each system. Long-range electrostatic interactions were calculated using Particle mesh Ewald method , and non-bonded interactions were calculated using the 10 Å cutoff distance. the bonds involving hydrogen atoms were restrained using the SHAKE algorithm . The temperature coupling 0.2 ps was used for temperature control. For each simulation, a 2 fs integration step was used.

**MM-GBSA calculations for binding free energies**

In the MM-GBSA method, the single-trajectory method was performed for the riboswitch +3′, 3′-cGAMP system . To address the energy contribution of each nucleotide of the riboswitch-ligand binding, the energy decompositions by per-nucleotide for the riboswitch +3′,3′-cGAMP and riboswitch + c-di-GMP systems were performed on the molecular mechanics, solvation free energies without consideration the entropies. For riboswitch +3′, 3′-cGAMP system, the binding free energy and per-residue decomposition energies were calculated using 500 snapshots from 60 ns to 80 ns of the simulation time.

**Principal component analysis**

Principle component analysis (PCA) is a statistical technique for dimensionality reduction of data . For PCA analysis, the covariance matrix of atomic coordinates was constructed from the MD simulation trajectories. The eigenvectors and the corresponding eigenvalues, which indicate the direction and amplitude of collective motion, were yielded by diagonalizing the matrix. The large eigenvalues represent the major motion. In our work, the first three principal components (PC1, PC2 and PC3) of 3′,3′-cGAMP riboswitch were extracted and analyzed from 60 ns to 80 ns of the simulation time of the apo riboswitch and riboswitch +3′, 3′-cGAMP systems.

**References**

1.	Essmann, U., A smooth particle mesh Ewald method. *J Chem Phys* **1995,** 103, (19), 8577-8593.

2.	Miyamoto, S.; Kollman, P. A., SETTLE: an analytical version of the SHAKE and RATTLE algorithm for rigid water models. *J comput chem* **1992,** 13, (8), 952-962.

3.	Case, D. A.; Darden, T.; Cheatham III, T. E.; Simmerling, C.; Wang, J.; Duke, R. E.; Luo, R.; Merz, K. M.; Pearlman, D. A.; Crowley, M.; Walker, R.; Zhang, W.; Wang, B.; Hayik, S.; Roitberg, A.; Seabra, G.; Wong, K.; Paesani, F.; Wu, X.; Brozell, S.; Tsui, V.; Gohlke, H.; Yang, L.; Tan, C.; Mongan, J.; Hornak, V.; Cui, G.; Beroza, P.; Mathews, D. H.; Schafmeister, C.; Ross, W. S.; Kollman, P. A., AMBER 9. *University of California, San Francisco* **2006**.

4.	Gohlke, H.; Kiel, C.; Case, D. A., Insights into protein-protein binding by binding free energy calculation and free energy decomposition for the Ras-Raf and Ras-RalGDS complexes. *J Mol Biol* **2003,** 330, (4), 891-914.

5.	Amadei, A.; Linssen, A.; Berendsen, H. J. C., Essential dynamics of proteins. *Proteins,***1993,** 17, (4), 412-425.

6.	Yamaguchi, H.; van Aalten, D. M. F.; Pinak, M.; Furukawa, A.; Osman, R., Essential dynamics of DNA containing a cis. syn cyclobutane thymine dimer lesion. *Nucleic Acids Res* **1998,** 26, (8), 1939-1946.

7.	Teeter, M. M.; Case, D. A., Harmonic and quasiharmonic descriptions of crambin. *J Phys Chem* **1990,** 94, (21), 8091-8097.

8.	Van Aalten, D.; Findlay, J.; Amadei, A.; Berendsen, H., Essential dynamics of the cellular retinol-binding protein evidence for ligand-induced conformational changes. *Protein Eng* **1995,** 8, (11), 1129-1135.

**Table S1.** MM-GBSA free energy (kcal·mol^-1^) components for the G20A-GEMM-I + 3′,3′-cGAMP model.

|  | G20A-GEMM-I + 3′,3′-cGAMP |
| --- | --- |
| Receptor: | class I GEMM riboswitch |
| Ligand: | 3′,3′-cGAMP |
| ΔE_ele_ | 1332.45 |
| ΔE_vdw_ | -72.57 |
| ΔG_np/solv_ | -7.64 |
| ΔG_pb/solv_ | -1281.83 |
| ΔG_np_ | -80.21 |
| ΔG_pb_ | 50.61 |
| ΔH | -29.60 |
| TΔS | -21.96 |
| ΔG_binding_ | -7.64 |
| ΔG_exp_ | -8.01 |
| k_d_ | 1.45 uM |

a: The values in parentheses are standard errors

Δ*G*_pb_ = Δ*E*_ele_ + Δ*G*_pb/solv_; Δ*G*_np_ = Δ*E*_vdw_ + Δ*G*_np/solv_; ΔH = Δ*G*_np_ + Δ*G*_pb_ + Δ*E*_int_; ΔG_binding_ = ΔH – TΔS;

ΔG_exp_= RTlnk_d_/(4.184*1000).

**Table S2.** MM-GBSA free energy (kcal·mol^-1^) components for three repeated molecular dynamics (MD) simulations (replica 1, replica 2, replica 3) of riboswitch+3′,3′-cGAMP, riboswitch+c-di-AMP, riboswitch+c-di-GMP and G20A-GEMM-I+3′,3′-cGAMP models.

|  | **riboswitch+3′,3′-cGAMP** | | | **riboswitch+c-di-GMP** | | | **riboswitch+c-di-AMP** | | | **G20A-GEMM-I+3′,3′-cGAMP** | | |
| --- | --- | --- | --- | --- | --- | --- | --- | --- | --- | --- | --- | --- |
| **Receptor** | **3′,3′-cGAMP riboswitch** | | | **3′,3′-cGAMP riboswitch** | | | **3′,3′-cGAMP riboswitch** | | | **G20A-GEMM-I riboswitch** | | |
| **Ligand** | **3′,3′-cGAMP** | | | **c-di-GMP** | | | **c-di-AMP** | | | **3′,3′-cGAMP** | | |
|  | **ΔH** | **TΔS** | **ΔG_binding_** | **ΔH** | **TΔS** | **ΔG_binding_** | **ΔH** | **TΔS** | **ΔG_binding_** | **ΔH** | **TΔS** | **ΔG_binding_** |
| replica 1 | -35.73 | -24.11 | -11.62 | -31.69 | -22.95 | -8.74 | -14.90 | -28.21 | 13.31 | -29.60 | -21.96 | -7.64 |
| replica 2 | -35.58 | -23.46 | -12.12 | -31.47 | -22.78 | -8.69 | -15.20 | -27.99 | 12.79 | -30.42 | -23.08 | -7.34 |
| replica 3 | -36.44 | -25.16 | -11.28 | -31.29 | -22.97 | -9.00 | -14.42 | -27.47 | 13.05 | -29.39 | -21.41 | -7.98 |

**Figure legends**

**Figure S1** Root-mean-square deviation **(**RMSD) values of all backbone atoms of the 3′,3′-cGAMP riboswitch with respect to the corresponding average structure for the simulations of apo riboswitch (green), riboswitch + c-di-GMP (blue), riboswitch + 3′,3′-cGAMP (magentas), riboswitch + c-di-AMP (violet) and G20A-GEMM-I + 3′,3′-cGAMP (cyan) models.

**Figure S2** The base-stacking interactions in the binding pocket for the average structure of the riboswitch + c-di-GMP system.

**Figure S3** The visual superposition for the average structures of the 3′,3′-cGAMP riboswitch for riboswitch + 3′,3′-cGAMP (magentas) and riboswitch + c-di-GMP (magentas)models. The P1 helix forming the fixed domain.

**Figure S4** Time-dependences of distances (**a**) between the J1/2 junction and ligand, (**b**) between the J2/3 junction and ligand, (**c**) between the J1/3 junction and ligand of the 3′,3′-cGAMP riboswitch for the riboswitch + 3′,3′-cGAMP (magentas) and riboswitch + c-di-AMP (violet) systems.

**Figure S5** Dynamical cross-correlation map from the last 10 ns of the 3′,3′-cGAMP riboswitch for the (**a**) riboswitch + 3′,3′-cGAMP, (**b**) riboswitch + c-di-GMP and (**c**) apo riboswitch models with the specific sub-regions squared in green and black. These key subregions squared in green and black involve in the direct and indirect allosteric communication networks, respectively.

**Figure S6** The visual superposition for the average structures of the 3′,3′-cGAMP riboswitch for the three repeated MD simulations (replica 1—white, replica 2:—yellow, replica 3—cyan) of (**a**) riboswitch + 3′,3′-cGAMP, (**b**) riboswitch + c-di-GMP and (**c**) apo riboswitch models.

Figure S1

Figure S2

Figure S3

(A)

(B)

(C)

Figure S4

(A)

(B)

(C)

Figure S5

(A)	(B)

(C)

Figure S6
